# MALDI-MS-Based Profiling of Serum Proteome: Detection of Changes Related to Progression of Cancer and Response to Anticancer Treatment

**DOI:** 10.1155/2012/926427

**Published:** 2012-07-30

**Authors:** Monika Pietrowska, Piotr Widłak

**Affiliations:** Center for Translational Research and Molecular Biology of Cancer, Maria Skłodowska Curie Memorial Cancer Center and Institute of Oncology Gliwice Branch, Wybrzeże Armii Krajowej 15, 44-101 Gliwice, Poland

## Abstract

Mass spectrometry-based analyses of the low-molecular-weight fraction of serum proteome allow identifying proteome profiles (signatures) that are potentially useful in detection and classification of cancer. Several published studies have shown that multipeptide signatures selected in numerical tests have potential values for diagnostics of different types of cancer. However due to apparent problems with standardization of methodological details, both experimental and computational, none of the proposed peptide signatures analyzed directly by MALDI/SELDI-ToF spectrometry has been approved for routine diagnostics. Noteworthy, several components of proposed cancer signatures, especially those characteristic for advanced cancer, were identified as fragments of blood proteins involved in the acute phase and inflammatory response. This indicated that among cancer biomarker candidates to be possibly identified by serum proteome profiling were rather those reflecting overall influence of a disease (and the therapy) upon the human organism, than products of cancer-specific genes. Current paper focuses on changes in serum proteome that are related to response of patient's organism to progressing malignancy and toxicity of anticancer treatment. In addition, several methodological issues that affect robustness and interlaboratory reproducibility of MS-based serum proteome profiling are discussed.

## 1. Cancer Markers and Clinical Proteomics 

Biological factors (e.g., proteins), whose status and/or quantity reflect the risk of a disease, severity of an illness, or the effects of therapy are called markers or biomarkers. In oncology, appropriately selected sets of markers can provide information about carcinogenic triggers to which the organism was exposed, detect early changes (hyperplasia, dysplasia) that appear prior to the occurrence of overt forms of cancer, as well as monitor efficacy and toxicity of the treatment. Such factors (i.e., potential biomarkers) are present in tumor tissues or body fluids, and encompass a wide variety of molecules, including transcription factors, cell-surface receptors, and secreted proteins. Several protein tumor markers have been used for decades in the traditional oncology for detection of cancer, for example, prostate cancer antigen (PSA) or cancer antigen 125 kD (CA125). In fact, effective tumor markers are in great demand since they have the potential to reduce cancer mortality rates by facilitating diagnosis of cancers at early stages and helping to plan tailored treatment. Cancer biomarkers can be divided into prognostic and predictive. Prognostic factors, which include tumor size, histological type of cancer, grade, and nodal status, allow the determination of prognosis regardless of a treatment. Currently list of prognostic molecular factors has been extended considerably including hormone receptors, markers of angiogenesis, and proliferation [1]. A predictive marker is a factor that indicates sensitivity or resistance to a specific therapy. The use of predictive markers appears increasingly relevant in contemporary cancer therapy as it allows for better identification of patients who will respond positively to selected therapy. Predictive molecular markers are used successfully in treatment of breast cancer [2]. Expression of estrogen and progesterone receptors can determine the benefits of hormone therapy, whilst the benefit of treatment with herceptin is determined by the expression of HER2. More recently predictive molecular markers are tested in many other malignancies, like somatic mutations of the EGF receptor gene used in the evaluation of the potential sensitivity of lung cancer patients to treatment with the receptor inhibitors [3]. However, approaching era of individualized treatment requires identification and validation of novel biomarkers. Hence proteomics, which is expected to deliver knowledge on novel cancer-related proteins, is among the most intensively developed field of molecular oncology.

Clinical proteomics is a division of proteomics focused on the analysis of proteome changes during development of the disease and during the progress of a therapy. This term was coined several years ago, *inter alia*, by Liotta and Petricoin [4]. The major objectives of clinical proteomics is to identify differences between proteomes of healthy and sick persons, relations between the state of the proteome and the degree of development of the disease, and changes initiated in response to the treatment. The primary aim of clinical proteomics is identification, characterization, and verification of protein biomarkers useful in the molecular diagnostics of the cancer [5–7]. There are generally two strategies for the detection of biomarkers using proteomics technologies. The first one is a “targeted” approach, which includes evaluation of candidates preselected using assumptions given from other traditional sources. The second strategy is a “nontargeted” *de novo* discovery approach, which could take advantage of full potential of proteomics. Most apparently both strategies can complement each other, and both have their positive and negative sides.

The object of clinical proteomics studies, similar to other types of medical diagnostics, can be either target tissue (or organ), where the disease develops, or a replacement tissue. Blood and its derivatives (serum or plasma) is the most common replacement tissue used in medical diagnostics. We expect that composition of blood reflects the presence and severity of disease. Given that serum (or plasma) is a very complex mixture and that hypothetical disease markers are present in low concentrations, testing the blood proteome requires properly selected and carefully optimized research tools. Current clinical proteomics is based on mass spectrometry (MS) tools, which different methods have successfully implemented also in analyses of blood proteome.

## 2. Implementation of Mass Spectrometry in ****Molecular Profiling of the Blood Proteome

Constantly improved mass spectrometric methods are widely used in proteomic studies. By using different techniques of MS it is possible to read the molecular weights of specific peptides and proteins in complex mixtures containing only pM of particular molecules. Depending on a type of analyte various techniques of ionization and separation of ions in the analyzers are used in the field. Two ionization methods: electrospray ionization (ESI) and matrix-assisted-laser(induced)desorption ionization (MALDI) are the most commonly used for protein analyses in current clinics-oriented studies. In MALDI, the analyte molecules (e.g., protein or peptide) are cocrystallized with matrix molecules that absorb UV light; cinnamic acid derivatives (such as sinnapinic acid, *α*-cyano-4-hydroxycinnamic) are the most frequently used for protein studies. As a result of matrix and analyte mixture irradiation molecules of analyte are protonated and desorbed from the crystals (matrix molecules serve as a source of protons). The resulting ions of the analyte are usually endowed with single protons ([M+H]^+^ molecular ions); the analyte molecules are not fragmented in normal conditions. MALDI spectrometers are used for the analysis of proteins and peptides with molecular weights ranging from 500 Da to as much as several hundred thousand Da [8, 9]. Similar principle of ionization is used in surface-enhanced laser desorption ionization (SELDI) spectrometers. In this type of spectrometers, the laser-beam-induced desorption of analyte molecules occurs from different types of surfaces, which specifically bind different fractions of proteins (such surfaces are coated with substances which are equivalent to chromatography deposits). MALDI (and SELDI) spectrometers are usually coupled with time of flight (ToF) ion analyzers. Registered time of flight of analyzed molecular ions (typically ranging from 0.01 to 1 ms) is directly proportional to the square root of *m/z* value and can be converted into mass of the analyte particles [8, 9]. Described method in its basic version allows on identification of characteristic features of the protein profiles of analyzed mixtures. However, MALDI-ToF spectrometry also allows the identification of proteins based on the amino acid sequence in polypeptide chains. Most typically, analyzed protein is processed enzymatically (e.g., by trypsin digestion), then selected peptides undergo further fragmentation in spectrometer, and sizes (*m/z* values) of fragmentary ions are determined in the so called tandem mass spectrometry (MS/MS). On the basis of fragmentary ion sizes, the appropriate computational analysis allows establishing of amino acids sequence in the peptide chain and identification of corresponding protein [10, 11]. 

The proteomics approach that takes into consideration characteristic features of the whole proteome but does not rely on particular protein, is called proteome pattern analysis or proteome profiling. In this approach specific proteome signatures are built based on several peptide/protein components, which are exemplified by ions registered at defined *m/z* values in the mass spectrum. Such proteome signatures (or patterns) can be used for further sample identification and classification (rev. [12–18]). The low-molecular-weight fraction of the blood proteome (up to 15,000 Da) appears to be a promising source of novel biomarkers of human diseases. Mass spectrometry methods, which allow characterization of this particular component of blood proteome, emerge as a potential and valuable tool of clinical proteomics and disease diagnostics. Numerous works have been published exploring the applicability of MALDI/SELDI-based profiling of the low-molecular-weight fraction of serum/plasma proteome for cancer diagnostics since the milestone paper was published by Petricoin and coworkers in 2002 [19]. These studies have shown that multi-peptide signatures selected in numerical tests have potential values for diagnostics of different types of cancer. Several major papers relevant for this topic are listed in Table 1. 

Authors of those works (as well as other works not quoted in the table) proposed multicomponent classifiers that could be used for detection and/or classification of cancer. This approach seems to be accepted as the first stage of the biomarker's discovery pipeline—identification of marker candidates. However due to apparent problems with standardization of methodological details, both experimental and computational, none of proposed serum peptide signatures analyzed directly by mass spectrometry has been approved for routine diagnostics yet. Noteworthy, several components of proposed cancer signatures, especially those for advanced cancer, were identified as fragments of blood proteins involved in the acute phase and inflammatory response [34, 48, 54]. This indicated that among cancer biomarker candidates to be possibly identified in serum proteome were rather those reflecting overall influence of a disease (and the therapy) upon the human organism, than cancer-specific molecules. Here, we aimed to point out the methodological issues that affect robustness of MS-based serum proteome profiling and to focus on two particular areas where such analyses seem to be the most rational and promising: assessment of cancer progression and monitoring of cancer treatment.

## 3. Problems Related to Standardization of ****Sample Processing and Data Analysis

One of the major challenges of MS-based serum/plasma proteomics is the high dynamic range in the abundance of particular blood proteins, exceeding 10 log scale [58]. The reproducibility and reliability of MS-based profiling of serum specimens is questionable because of problems with identification of low-intensity components in the background of highly abundant “housekeeping” proteins (e.g., albumins and immunoglobulins) [26, 59]. The fragments of highly abundant proteins dominate the peptide profiles of plasma specimens [39, 60]. In fact, most studies demonstrated that candidate biomarkers represent fragments of high-abundance serum proteins, rather than specific tumor-associated gene product (rev in [11]). In theory, mass spectrometer should detect the presence of all types of molecules present in the specimen. However, MS is not a quantitative method, and the intensity of registered ion does not reflect its actual concentration in the sample. The major difficulties associated with the measurement of potential biomarkers in serum/plasma samples are: (i) low abundance of particular components in the specimen, (ii) masking the candidate by abundant proteins (such albumin and immunoglobulins), and (iii) the candidate stability. 

The presence of albumin (representing approximately 60% of all serum proteins, 50 mg/mL) is the major factor changing the yield of ionization and affecting the ability to register low abundance serum components [61]. Such influence on measurement of less abundant molecules is named suppression effect. Preprocessing of the serum sample and removal of albumin (and other high-molecular-weight proteins) from the specimen before the analysis should be always considered. The depletion of highly abundant proteins form the serum specimen could be achieved in different ways. Among them are: immunoaffinity depletion using custom made or commercially available columns (e.g.: Qproteome Albumin/IgG Depletion Kit (Qiagen), Montage Albumin Deplete Kit (Millipore), or MARS columns/spin cartridges (Agilent)), fractionation by size exclusion chromatography, ion exchange chromatography, and/or isoelectric focusing. Ultrafiltration of serum (and other body fluids), where components of high molecular weight are retained while the low molecular weight proteins/peptides pass through the membrane, is among the most popular methods of sample preprocessing [62]. However, even though removal/depletion of the most abundant proteins increases coverage of serum components [63], depletion of albumin might be risky because several low-molecular-weight components of the blood are attached to this carrier protein and could be codepleted from the specimen [64]. Several methods, including size exclusion ultra-filtration under denaturation conditions [65], continuous elution denaturing electrophoresis [66], or fractionation of serum/plasma by nanopores [67, 68] have been proposed to omit this problem. In many applications, serum is diluted with solution of a denaturing factor acetonitrile (final v/v concentration 20–25%). The presence of acetonitrile affects protein/protein interactions and releases low-molecular-weight peptides/proteins associated with albumin or other carrier proteins, which significantly increases amount of proteins passing through the ultrafiltration membrane [62]. Conditions of ultrafiltration (e.g., selection of size of pores in the membrane, dilution of specimen, and concentration of acetonitrile) largely affect composition of the filtrate and has to be carefully considered [62, 69–71]. Another problem related to serum sample processing and storage is stability of analyzed proteins/peptides. First of all, blood for isolation of serum should be collected, transported, stored, and processed in conditions that minimize sample degradation by proteases and contamination (e.g., by bacteria) [72, 73]. Recently hydrogel nanoparticles have been applied in biomarker purification and preservation (rev. [66–68, 74]). Hydrogel particles containing an affinity bait could be used for: (i) rapid one-step sequestration of the low-molecular-weight fraction of serum proteins and metabolites; (ii) removal of the target molecules from sample; (iii) protection of proteins from enzymatic degradation; (iv) concentration of the target protein. This technology might increase the detection limit of mass spectrometry and immunoassays by >100 fold [75]. Depending on selected method of serum sample processing, including or excluding depletion of the high-molecular-weight proteins, different low-molecular-weight peptide signatures (classifiers) should be expected even though overall power of resulting classification might be similar [42].

Proteomics studies generate high-dimensional data sets where traditional statistical and computational methods are not sufficient for analyses, hence novel “nonstandard” mathematical approaches are frequently required. A typical proteomic analysis of spectral data consists of three steps: (i) preprocessing of data, (ii) identification of spectral components, and (iii) statistical analyses and classification based on identified components. The first step includes: “smoothing” of the spectrum, removing the baseline, normalization, and averaging of technical replicates. The simplest method of spectra “smoothing” is averaging among several neighboring points. The baseline correction flattens specific noise named baseline. Interpolation is performed to standardize points on the *m/z* axis among all spectra. Normalization is used for scaling the spectra—the most frequently used algorithms base on total ion current (TIC) value or constant noise. All procedures reduce dimensionality of data and improve quality of further analyses. There are several algorithms accepted for procedures and most of proteomics studies used comparable methods for spectra preprocessing. However, there is no single protocol generally accepted as the standard. In fact some of the key parameters are tuned and adjusted for particular analyses, and type and order of preprocessing steps may significantly differ among different analyses [76–78]. There are two general types of approaches allowing comparison of multiple spectra. One of them bases on comparing the signal in successive measurement points (called point-to-point analysis). The second one uses identification and matching of spectral components. This approach usually starts from peak detection. The aim of peak detection procedures is to find peaks describing the composition of spectra, which is followed by alignment of peaks identified in different spectra. Most of algorithms used for peak detection identify local maxima and minima; local maxima are classified as peaks only when they have signal-to-noise ratio above a given threshold [79]. Alternative procedure for identification of spectral components bases on modeling of spectra as a sum of Gaussian components, then the model is adjusted to experimental data. Such procedure avoids artifacts linked with the methods of point-to-point and peak alignment, and facilitates quantitative and statistical analysis [43]. The objective of the last step of spectral data analysis is to find patterns in the data sets and to classify samples. Many of statistical and analytical algorithms and tools developed for functional genomics are being used in proteomics. Different combinations of unsupervised and supervised clustering, machine learning, pattern recognition, statistical analysis, modeling techniques, and database mining are used in the field. Some researchers use custom-build protocols and algorithms while others base on commercially available software for spectra analysis (e.g., ClinProTools (Bruker Daltonics), Spectrolyzer (MedicWave)). All these computational tools have to be tuned and optimized for specific applications and datasets, which apparently add diversity to these protocols.

Many different experimental and computational procedures are used in the field for sample preprocessing and data analysis. All these procedures are modified and adjusted for specific objectives and sample or data sets to deliver optimal results. However, the major drawback of the situation is the lack of “golden standard” in sample and data processing, which makes impossible direct comparison of peptide signatures delivered in different studies of blood proteome of cancer patients. Hence, comparative analysis and interpretation of such data requires caution and should be preferably performed based on sequence-identified components of peptide profiles.

## 4. Features of Serum Proteome Profiles Reflect Stage and Progression of a Cancer Disease

Precise and adequate assessment of degree of tumor progression is a key factor for proper selection of the optimal treatment. However, the traditional anatomy-based classification of cancer stages, the so-called TNM classification [80–83], turns out to be imperfect and in many cases insufficient for planning of modern-tailored therapy. Therefore, preliminary trials and attempts are implemented in oncology during last years to supplement traditional classification of cancer stages with novel measures and parameters. Some of these attempts base on precise measurement of tumor volume and assessment of clonogenic cells number by functional/molecular imaging methods [84, 85]. Another obvious direction of research is aimed at identification of novel molecular factors that correlate with degree of tumor progression, and could be used as biomarkers complementary with anatomical/volumetric methods for the classification of solid tumors. Currently the dominant diagnostic tools for the analysis of protein markers in the blood bases on immunological methods (e.g., ELISA). In clinical practice immune-based methods are used to measure the number of protein markers, which levels in blood correlate with the progression of cancer. Such markers include protein CA125, CEA, PSA, betaHCG, AFP, and CA19-9. Importantly, these markers, although used routinely, have limited predictive value and their increased levels are observed primarily in patients with heavily advanced cancer (usually in the stage of spreading) [86–88]. Detectability of those markers mostly in advanced cancer cases apparently limits their use for more refined assessment of cancer progression. Hence, identification of new markers with potential applicability in cancer staging is an important task for cancer proteomics. Among several important mechanisms involved in tumor biology, which could serve as a source of potential biomarker for assessing progression of cancer, there are immune response and inflammatory reactions. In general immunity and inflammation processes related to immune response have dual role in malignant tumors. The immune system plays an important role in anticancer protection. However, inflammation could also enhance tumor growth when become a chronic reaction. On the other hand, a growing malignant tumor often maintains the tissue in the inflammatory state. Lymphocytes and other components of the immune system present in tumor are often immunosuppressed (i.e., unable to destroy the cancer cells), but still secrete growth factors and cytokines that contribute to tumor growth (rev. [89–93]). Chronic inflammatory reactions are frequently observed in cancer patients and their escalation putatively correlates with progression of malignancy. This is due to stimulation of the immune system and tissue damage in the course of the disease. A sensitive marker of chronic and acute inflammatory processes are acute phase proteins. Their levels in the blood increase in response to tissue damage, infection, and other stimulants of the inflammatory response (e.g., cytokines). The most important acute phase proteins are: *α*1-proteinase inhibitor, *α*1-acid glycoprotein, serum amyloid A (SAA), haptoglobin, ceruloplasmin, *α*2-macroglobulin, and C-reactive protein (CRP). Interestingly, many acute-phase proteins were identified as potential markers of the process of carcinogenesis [94–96]. An important inflammation-related protein, which increased abundance in blood is characteristic for cancer patients is serum amyloid A (SAA). SAA is an acute-phase apolipoprotein typically induced in liver in response to inflammatory stimuli. However, increased expression of SAA was also observed during tumorogenesis, and elevated serum level of this protein was a general feature of progressive and metastatic cancer cases. For example, the presence of SAA was demonstrated in 55% of ovarian cancer cases and only in 6% of healthy individuals [97, 98]. Other authors have shown nearly 20-fold increase in SAA in the case of lung cancer patients compared to healthy subjects, and SAA levels in patients with poor prognosis (survival less than 5 years) was 2-fold higher than in patients with a better prognosis [99]. Thus SAA was proposed to be a prognostic cancer marker [88]. 

Several components of cancer signatures proposed in MS-based serum proteome studies, especially those characteristic for advanced cancer, were identified as fragments of blood proteins involved in the acute phase and inflammatory response (rev. in [90, 100]). This indicated that among cancer biomarker candidates to be found by this approach were those reflecting overall influence of a disease upon the human organism. Most profiling studies have demonstrated that candidate biomarkers represent mere fragments of high-abundance serum proteins, rather than specific tumor-associated gene products. The contribution in proposed cancer signatures of components specific for particular types of malignancies and “nonspecific” components related to general response of the organism was not established so far. Comparative analysis of serum peptides detected in samples from healthy persons and breast, bladder, or prostate cancer patients allowed identification of cancer-specific features, yet some differentiating peptides were common for all three cancer signatures (e.g., fragments of fibrinopeptide A). Another coagulation-related candidate biomarker identified by serum profiling of cancer patients is 2664 Da fibrinogen alpha-chain fragment. This peptide has significantly increased abundance in blood of patients with oral cancer when tested using MALDI spectrometry [28]. Importantly, increased level of fibrinogen alpha-chain was previously reported in blood of patients with gastric cancer, where it was correlated with progression of the malignancy [101], and melanoma patients [102]. Another cancer-specific marker identified by MS-based serum proteome profiling was SAA. Increased levels of fragments of SAA (~11,5 kDa and ~11,7 kDa) were detected by MALDI/SELDI spectrometry in serum of patients with different types of advanced cancer, for example, ovarian cancer [98], prostate cancer [48], renal cancer [54, 103], colon cancer [104], and lung cancer [34, 95, 105]. All these reports indicate collectively that SAA1 fragments are indeed cancer-stage-specific but not cancer-type-specific components of cancer peptide signatures.

## 5. Features of Serum Proteome Profiles Reflect Response of Patients' Organism to Anticancer Therapy

A few studies have focused on application of MS-based serum proteome profiling to identify possible therapy-related features that could be used for monitoring of progression, efficacy, and toxicity of the treatment. Some of these works were related to analysis of serum/plasma of patients undergoing chemotherapy because of breast cancer. SELDI-ToF analysis of the plasma proteome of breast cancer patients who underwent paclitaxel-based neoadjuvant treatment revealed one peptide (*m/z*  =  2790 Da) that specifically increased in its abundance, yet the presence of this peptide did not correlate with the outcome of therapy [40]. Similar analysis of the serum proteome of patients infused with docetaxel revealed two peptides (*m/z*  =  7790 and 9285 Da), which changed their abundances in response to the treatment. These peptides were identified as kininogen and apolipoprotein A2, respectively [41]. Such taxane-induced changes were detected in samples collected just few days (or hours) after the exposure to the drug, and apparently reflected short-tem effects of the treatment. There are also studies that addressed long-term effects related to the treatment of breast cancer patients. In one small-scale study 16 paired serum samples collected from breast cancer patients before the treatment and posttreatment (6–12 months after surgery and at least one month after the end of adjuvant therapy) were analyzed using SELDI-ToF; the treatment scheme was heterogenous in this group and based on surgery alone, or surgery supplemented with neoadjuvant chemotherapy or adjuvant chemo/radiotherapy. It was found that three peptides (*m/z*  =  2276, 4892, and 6194 Da) increased their abundance in serum collected posttreatment. Noteworthy, both pretreatment and posttreatment samples retained specific features of mass profiles that differentiated them from serum samples collected from healthy donors [106]. We have performed MALDI-based comparative analysis of the low-molecular-weight fraction of serum proteome in a larger group (70 person) of patients diagnosed at early stages of breast cancer and treated with surgery either independently or in combination with adjuvant radio/chemotherapy; samples collected before the start of therapy, after the surgical resection of tumors and one year after the end of therapy were compared. We found that surgical resection of tumors did not have an immediate effect on serum proteome profiles. On the other hand, significant long-term effects were observed one year after the end of basic treatment. Moreover, the significant differences were found primarily in the subgroup of patients treated with adjuvant therapy, but not in the subgroup subjected only to surgery. This suggests that the observed changes reflect overall responses of the patients' organisms to the toxic effects of adjuvant radio/chemotherapy. In line with this hypothesis we detected two serum peptides (registered *m/z* values 2184 and 5403 Da) whose changes correlated significantly with the type of treatment employed (their abundances decreased after adjuvant therapy, but increased in patients treated only with surgery) [42]. There is an intriguing possibility whether therapy-related changes in serum proteome correlated with clinical outcome. An SELDI-based study performed in patients treated with neoadjuvant radiochemotherapy of rectal cancer revealed that specific features of serum proteome profiles observed in the course of the treatment discriminated patients with good and poor histological response to the therapy [107]. 

There are also a few works focusing on changes in serum proteome composition induced specifically by exposure of cancer patients to ionizing radiation. SELDI-based proteome profiling was applied in comparative analysis of serum samples collected before and during or directly after radiotherapy of cancer patients. Analyzed group consisted of 68 patients treated with radiation due to 18 different malignancies, who received total doses in a range of 1.5 to 86.4 Gy (median 48.6 Gy), with the time interval between consecutive blood samples in a range of 1 to 55 days (median 35 days; second sample was usually collected in the latter part of the treatment). In spite of a very heterogenous material the authors identified differences between preexposure and postexposure samples. Registered serum proteome profiles were used to build multicomponent classifiers that differentiated unexposed and exposed samples, as well as samples collected from patients exposed to higher and lower doses of radiation [108]. We performed MALDI-based analysis of the low-molecular-weight fraction of serum proteome of patients treated with radical radiotherapy alone due to larynx cancer (total doses ranging from 51 to 72 Gy). Three consecutive blood samples were collected from each of 46 patients: before the start, 2 weeks after the start, and 1-2 months after the end of radiotherapy. Significant differences were not found between serum samples collected before the start and during radiotherapy. In marked contrast, however, numerous spectral components showed significantly changed abundances in samples collected after the end of radiotherapy. This report showed for the first time that radiation-related changes in abundance of serum peptides could be detected several weeks after the irradiation. In addition, the abundance of certain serum peptides was associated with a dose of radiation received by patients. This indicated that features of serum proteome could be a useful retrospective marker of exposure to ionizing radiation and that multi-component serum peptide signatures determined by MS could have applicability in radiation dosimetry [35].

All these data indicate collectively high potential applicability of MS-based serum proteome profiling in monitoring the response of cancer patients to chemo- and radiotherapy. Changes in abundance of particular low-molecular-weight components of blood proteome observed in the course of anticancer therapy could reflect several different processes, including direct toxic effects of the treatment, healing of toxic reactions, and effects related to removal of a tumor. Not surprisingly among serum proteins that changed their abundance during the treatment several were identified as acute-phase proteins. This confirmed that processes reflecting the general reaction of patient's organism to the malignancy and its treatment were major targets of MS-based profiling of the low-molecular-weight fraction of serum proteome.

## 6. Conclusions

In spite of tens of published studies the real potential of MALDI/SELDI-based serum proteome profiling in clinical diagnostics is not clearly defined so far. Even though these studies have showed that multipeptide signatures selected in numerical tests have some potential value for classification and differentiation of cancer, none of proposed serum peptide signatures has been approved for routine diagnostics. Lack of standardization of methodological details, both experimental and computational, is an important problem in MS-based blood proteome profiling. It appears that guidelines for standardization of these multiparametric analyses should be generally accepted in the field and that identified marker candidates need to be confirmed in multicenter bias-free prospective validation studies. 

## Figures and Tables

**Table 1:** Examples of MS applications to analyze the serum proteome in the diagnosis of cancer.

Type of cancer	Publications
Ovarian cancer	[19–24]
Head and neck cancer	[25–36]
Breast cancer	[37–44]
Prostate cancer	[39, 45–48]
Uterus cancer	[49]
Lung cancer	[50]
Colon cancer	[51]
Pancreatic cancer	[52]
Thyroid cancer	[53]
Renal cell carcinoma	[54]
Bladder cancer	[39]
Liver cancer	[55]
Leukemia	[56, 57]
